# Cross Talk between Adipose Tissue and Placenta in Obese and Gestational Diabetes Mellitus Pregnancies *via* Exosomes

**DOI:** 10.3389/fendo.2017.00239

**Published:** 2017-09-27

**Authors:** Nanthini Jayabalan, Soumyalekshmi Nair, Zarin Nuzhat, Gregory E. Rice, Felipe A. Zuñiga, Luis Sobrevia, Andrea Leiva, Carlos Sanhueza, Jaime Agustín Gutiérrez, Martha Lappas, Dilys Jane Freeman, Carlos Salomon

**Affiliations:** ^1^Exosome Biology Laboratory, Centre for Clinical Diagnostics, University of Queensland Centre for Clinical Research, Royal Brisbane and Women’s Hospital, The University of Queensland, Brisbane, QLD, Australia; ^2^Maternal-Fetal Medicine, Department of Obstetrics and Gynecology, Ochsner Clinic Foundation, New Orleans, LA, United States; ^3^Faculty of Pharmacy, Department of Clinical Biochemistry and Immunology, University of Concepción, Concepción, Chile; ^4^Cellular Signaling and Differentiation Laboratory (CSDL), Medical Technology School, Health Sciences Faculty, Universidad San Sebastian, Santiago, Chile; ^5^Cellular and Molecular Physiology Laboratory (CMPL), Division of Obstetrics and Gynaecology, Faculty of Medicine, School of Medicine, Pontificia Universidad Católica de Chile, Santiago, Chile; ^6^University of Queensland Centre for Clinical Research, Royal Brisbane and Women’s Hospital, The University of Queensland, Brisbane, QLD, Australia; ^7^Faculty of Pharmacy, Department of Physiology, Universidad de Sevilla, Seville, Spain; ^8^Obstetrics, Nutrition and Endocrinology Group, Department of Obstetrics and Gynaecology, University of Melbourne, Melbourne, VIC, Australia; ^9^Mercy Perinatal Research Centre, Mercy Hospital for Women, Heidelberg, VIC, Australia; ^10^Institute of Cardiovascular and Medical Sciences, University of Glasgow, Glasgow, United Kingdom; ^11^Mater Research Institute-University of Queensland, Translational Research Institute, Woolloongabba, QLD, Australia

**Keywords:** adipose tissue, extracellular vesicles, adipose tissue-derived exosomes, obesity, gestational diabetes

## Abstract

Obesity is an important public health issue worldwide, where it is commonly associated with the development of metabolic disorders, especially insulin resistance (IR). Maternal obesity is associated with an increased risk of pregnancy complications, especially gestational diabetes mellitus (GDM). Metabolism is a vital process for energy production and the maintenance of essential cellular functions. Excess energy storage is predominantly regulated by the adipose tissue. Primarily made up of adipocytes, adipose tissue acts as the body’s major energy reservoir. The role of adipose tissue, however, is not restricted to a “bag of fat.” The adipose tissue is an endocrine organ, secreting various adipokines, enzymes, growth factors, and hormones that take part in glucose and lipid metabolism. In obesity, the greater portion of the adipose tissue comprises fat, and there is increased pro-inflammatory cytokine secretion, macrophage infiltration, and reduced insulin sensitivity. Obesity contributes to systemic IR and its associated metabolic complications. Similar to adipose tissue, the placenta is also an endocrine organ. During pregnancy, the placenta secretes various molecules to maintain pregnancy physiology. In addition, the placenta plays an important role in metabolism and exchange of nutrients between mother and fetus. Inflammation at the placenta may contribute to the severity of maternal IR and her likelihood of developing GDM and may also mediate the adverse consequences of obesity and GDM on the fetus. Interestingly, studies on maternal insulin sensitivity and secretion of placental hormones have not shown a positive correlation between these phenomena. Recently, a great interest in the field of extracellular vesicles (EVs) has been observed in the literature. EVs are produced by a wide range of cells and are present in all biological fluids. EVs are involved in cell-to-cell communication. Recent evidence points to an association between adipose tissue-derived EVs and metabolic syndrome in obesity. In this review, we will discuss the changes in human placenta and adipose tissue in GDM and obesity and summarize the findings regarding the role of adipose tissue and placenta-derived EVs, with an emphasis on exosomes in obesity, and the contribution of obesity to the development of GDM.

## Introduction

Globally, the incidence of obesity has increased tremendously over the years and become a significant and challenging issue to be addressed (1). Obesity is defined as a body mass index (BMI) of ≥30 kg/m^2^. Obesity-related diseases and health problems are wide-ranging and pose a substantial threat to healthcare services. Cardiovascular diseases (CVD), stroke, high blood pressure, type 2 diabetes (T2D), and certain forms of cancers are among the harmful effects of obesity (2). According to the Centres for Disease Control and Prevention (CDC), between 2011 and 2014 over 36% of adults were considered obese and the prevalence was much higher in women (38.3%) compared to men (34.3%). Obese women have a higher risk of obstetric complications, especially gestational diabetes mellitus (GDM) (3), with population-based studies demonstrating that approximately 50% of GDM cases are caused by obesity (4). GDM is characterized as hyperinsulinemia and hyperglycemia in the maternal systemic circulation during gestation (5). Globally, GDM affects approximately 9–15% of all pregnancies and Australia is no exception (6). Although gestational glucose intolerance returns to normal postnatally, women with a history of GDM have a greater risk of developing T2D later in life. In addition, their babies are at an increased risk of becoming overweight with serious metabolic problems in their adult life (7). In fact, a female child of a GDM mother faces a higher possibility of developing GDM during her subsequent confinement, and the cycle continues (8). The need for early diagnosis of GDM is pressing given that the oral glucose tolerance test is the only available gold standard to diagnose GDM at 24–28 weeks of pregnancy (9). However, GDM pathology is mostly only established by the second trimester of pregnancy, meaning that the potential to reverse this condition is limited (10). Thus, understanding its pathophysiology is important in optimizing a treatment plan and achieving an optimal outcome.

Adipose tissue plays an important role in the development of obesity and its related diseases. An increase in the number and size of adipocytes are among the changes that can be observed in obesity (11). Besides these histological changes, adipose tissue undergoes functional changes in obesity that include deregulated secretion of pro-inflammatory cytokines (“adipocytokines”) which contribute to the development of insulin resistance (IR) (12).

Recently, it has been shown that adipose tissue membrane-derived vesicles termed EVs (13, 14) are produced. EVs have been extensively studied for their involvement in intercellular communication which usually occurs *via* the transfer of bioactive molecules, such as proteins, lipids, and RNAs, from their parent cells (15–18). Intercellular communication is an essential part of body processes and they allow for the proper coordination of biological functions as well as enabling the progression of various diseases. The role of adipose tissue EVs may, thus, contribute to the pathophysiology of GDM, particularly in those cases that are also complicated by obesity.

## Extracellular Vesicles (EVs)

Extracellular vesicles are membrane-derived vesicles, playing key roles in cell-to-cell communication and conveying molecular signals to cells at proximal as well as distal locations (19, 20). Initially, EVs were regarded as “debris” generated by cells, however, substantial research in this area revealed that these membrane-derived vesicles interact with their target cells and perform crucial modulatory functions in their biological signaling (21–23). EVs comprise a heterogeneous group of vesicles, classified on the basis of their origin, morphology and mode of release into the extracellular milieu. There are three major vesicle populations, namely apoptotic bodies, microvesicles (MV), and exosomes. Apoptotic bodies (0.8–5 µm in diameter) are released from cells undergoing programmed cell death (24). MVs (0.1–0.35 µm in diameter), also known as ectosomes, originate from external budding of the plasma membrane (25, 26). The main focus of the current review are the “exosomes” which are nano-sized vesicles (50–120 nm in diameter) formed from inward budding of late endosomal structures called multivesicular bodies (MVB) and exocytosed *via* fusion of MVBs with the plasma membrane (26, 27). Exosomes are like “fingerprints,” uniquely reflecting the phenotype of their parent cell. Emerging research reveals their key role in harmonizing and regulating molecular pathways in their recipient cells, shedding light on the pathophysiological mechanisms in various diseases. The initial biogenesis and release of these endocytic nano-sized vesicles are the initial and most critical steps in the exosome signaling pathway for exerting their biological functions in target cells.

### Exosomes Characteristics and Biogenesis

Exosomes are present in almost all biological fluids and have been isolated from a variety of these fluids as well as from cell culture media (28–39). Exosome isolation is an extensive area of research and can be performed by various methods, including differential centrifugation, density gradient centrifugation, size exclusion chromatography, filtration, polymer-based precipitation, immunological separation, and isolation by sieving (40, 41). Each method has inherent advantages and disadvantages depending on the downstream applications of the isolated exosomes (42–44).

Exosomes have been described as having a “cup-shaped” morphology in electron microscopy. In addition, exosomes equilibrate at densities between 1.13 and 1.19 g/ml on continuous sucrose gradients (39). Identification of exosome specific markers has a vital role in characterizing exosomes and differentiating them from other EVs. These markers are proteins that are specific to the endosomal pathway. These include proteins related to MVB biogenesis, such as Tsg101, Alix, and tetraspanins (CD-63, CD-9, and CD-81); membrane fusion proteins, such as RAB GTPases and Annexins; and signaling molecules, such as cell adhesion molecules, growth factor receptors, and heat shock protein (HSP)-70 and HSP-90 (45–47). The endosomal sorting complex required for the transport (ESCRT) pathway facilitates membrane remodeling and has been implicated in the formation of intraluminal vesicles (48). An ESCRT-independent pathway has also been described as MVBs can be produced in the absence of all four ESCRT complex subunits (49, 50). Finally, the release of exosomes to the extracellular milieu occurs by the fusion of the matured MVB with the plasma membrane, mediated by Rab GTPases (51, 52). Exosomes are enclosed by the phospholipid bilayer of their parent cell and contain a small fraction of cytoplasm taken up from their cell of origin. Hence, exosomes are loaded with a wide variety of molecules, including proteins, RNAs, lipids, and fragments of genomic DNA (53–55) that are present in the parent cell. Exosomes, when released into the extracellular space, can act proximally but can also enter the circulation and cross physiological barriers, eliciting their actions at distal locations (30, 56, 57). The biological function of exosomes relies primarily on the interaction between the exosome and its target cell.

### Exosome Signalling

In order to exert their biological functions, exosomes must be taken up and release their contents into the new host cells. Understanding of the mechanisms by which the signals are processed by target cells is still at its infancy. However, a number of key discoveries have been made that aid the understanding of exosome uptake and signaling in the target cells.

Endocytosis of exosomes is *via* the exosomal trafficking pathway. The endocytosis process can occur *via* phagocytosis (58) or receptor and raft-mediated endocytosis (59, 60). The phagocytosis mechanism occurs mainly in phagocytic cells. Feng et al. (58) demonstrated that RAW 264.7 macrophages cells effectively internalized exosomes derived from K562 and MT4 cell lines. The internalization was actin-mediated and dependent on phosphatidylinositol 3-kinase (PI3K) and dynamin2. Similarly, Tian et al. (61) showed that pancreatic cancer cells internalized exosomes and the engulfed exosomes were shown to merge with endosomes of the recipient cell and potentially transported to neighboring cells (62).

By contrast, receptor-mediated endocytosis can occur *via* the classical or non-classical pathway. The former occurs *via* caveolin or clathrin membrane proteins. The exosomes derived from virus-infected cells were demonstrated to be internalized by target cells *via* caveolin-dependent endocytosis. Knockdown of the CAV1 gene lead to significantly reduced exosome uptake, proving caveolin-mediated endocytosis (63). Bone marrow-derived mesenchymal stromal cells were shown to take up PC12 cell-derived exosomes *via* clathrin-mediated endocytosis and contributed to alterations in gene expression through the transfer of miR-21 (64). Similarly, an investigation of uptake of macrophage-derived exosomes by the BeWo cell line and human trophoblast cells showed that uptake is an endocytic process mediated by clathrin (62). In addition, the uptake of exosomes induced secretion of pro-inflammatory cytokines by the placental cells. This study demonstrates a change in placental phenotype induced by exosomes.

On the other hand, the non-classical endocytic uptake of exosomes can occur independent of membrane proteins. It has been reported that exosome uptake by glioblastoma cells occur *via* lipid raft-mediated endocytosis and is dependent on extracellular signal-regulated kinase-1/2 and HSP27 (60).

Another form of exosome–cell interaction is the adhesion of exosomes to a potential docking site found on target cells. This mode of interaction is facilitated by the presence of transmembrane proteins on the surface of the exosomes. Dendritic cell-derived exosomes express intercellular adhesion molecule-1, major histocompatibility complex, and co-stimulatory molecules which enable the exosomes to interact with target cells *via* their respective signaling receptors (65–67).

By interacting with the recipient cells, exosomes potentially transfer their cargo which is capable of regulating the biological function of the recipient cells. This then orchestrates diverse signaling pathways and mediates a broad range of physiological and pathological conditions. Cellular responses to the microenvironment have a decisive role in determining the concentration and content of exosomes. This has opened up new avenues for biomarker discovery and therapeutic interventions (68–70).

### Trafficking of Exosomes and Exosomal MicroRNA (miRNA) between Cells

All cell types in the human body secrete exosomes, including adipose tissue, liver, pancreas, skeletal muscle and placenta during pregnancy. Exosomes released from metabolically active cells could effectively coordinate communication between tissues and initiate metabolic reprogramming in the end target organs. This represents a potential platform for the progression of metabolic disease.

Co-incubation of differentiated C2C12 (muscle cells) with exosomes isolated from C2C12 pre-treated with fatty acid (FA) induced alteration in the gene and proteins expressions in the muscle cells. This indicates that exosomes transfer the effects of FA between the muscle cells and this could disrupt homeostasis and lead to IR in muscle cells. In the same study, C2C12-derived exosomes were injected into mice and were found distributed in various tissues, including metabolic tissues (71).

By utilizing pancreatic cancer-derived exosomes, Wang et al. (72) demonstrated that the exosomes entered skeletal muscle cells, initiated lipidosis, and inhibited glucose uptake. In addition, the exosomes downregulated the insulin and PI3K/Akt signaling pathway and impaired the activity of their downstream target, glucose transporter (GLUT)4. In a reciprocal experiment, it was shown that exosomes isolated from skeletal muscle of high fat diet fed mice were taken up by MIN6B1 cells and mouse islets. The release of the exosomal miRNA changed the expression of mRNAs and genes of the MIN6B1 cells as well as inducing the proliferation of MIN6B1 and islets (73). This suggests that skeletal muscle-derived exosomes could potentially provoke IR in distant cells *via* exosomes.

Similarly, IR in muscle cells was observed after co-incubation with macrophages treated with adipose tissue-derived exosomes (74). This suggests that adipose tissue-derived exosomes could act as a mediator for the onset of metabolic disease. The studies reviewed here suggest that exosomes secreted by cells from metabolic tissues can coordinate metabolism among tissues and be an effective initiator of the onset of metabolic disease, including diabetes and GDM during pregnancy.

Although exosomes contained a wide variety of molecules, miRNAs has been the center of attention mainly due to its role in regulating gene expression. The exosomal miRNAs are trafficked from their parent cells and the exosomal profile varies according to the physiological conditions of their parent cells. The chromosome 19 miRNA cluster (C19MC) is a unique group of 58 miRNAs exclusively expressed in the human placenta and in undifferentiated cells (75, 76). Growing evidence highlights the presence of these placental-specific miRNAs in exosomes (77, 78). Luo et al. (79) demonstrated that release of C19MC miRNAs is *via* exosomes and one of the C19MC-encoded miRNA is involved in tumor necrosis factor (TNF)-α signal transduction. miRNA profiling of whole blood and blood-derived exosomes obtained from patients with metabolic syndrome detected similar expression of miR-17, miR-197, miR-509-5p, miR-92a, and miR-320a (80). However, the proportion of exosomal miRNAs is higher than that in their parent cells (81). Interestingly, the exosomal miRNA profile can differ from those of their parent cells (15). The analysis of liver tissue and exosomes (and MV) isolated from a non-alcoholic fatty liver disease (NAFLD) animal model showed enrichment of miR-122 and miR-192 in the vesicles and relative deficiency in the tissue (82).

Hence, the shuttling of miRNAs from parent cells to exosomes involves selective mechanisms. However, there is a paucity of data defining the selective compartmentalization of miRNAs into exosomes.

### The Microenvironment Modulates Exosome Profile

Although exosomes are produced from cells in a constitutive manner, pathophysiological conditions and stress can modulate exosome biogenesis and release. Recent research provides insight into the selective sorting of proteins and miRNAs into exosomes in conditions of physiological change or pathological stimuli, leading to modification of exosome proteome and RNA profile and, thus, mirroring the microenvironment in the parent cell (83–85).

Hypoxia or low oxygen tension is a stress-induced physiological condition and a classical phenotype in several diseases, such as ischemic CVD, malignancies of diverse origins, obesity, preeclampsia, and physiological challenges such as pregnancy. Hypoxia induces the activation of hypoxia-inducible factor (HIF) which is a key mediator in the cellular adaptation to low oxygen concentrations. HIF, a major modulator of exosome biogenesis and HIF-mediated intercellular exosome signaling, has been identified in a vast array of physiological and pathological conditions (86, 87). Increased endothelial cell migration and angiogenesis is central to the cellular hypoxic response. Increasing evidence suggests the potential relevance of exosomes in mediating these vascular changes. Angiogenic ability has been attributed to exosomes derived from aggressive tumors.

The crucial role of exosomes in remodeling the hypoxia-induced tumor microenvironment has been well elucidated (85, 88–91). Hypoxic tumor exosomes are loaded with unique proteins and have an enhanced capacity for invasiveness, stemness, and tumor progression (87, 92). Hypoxia-induced endothelial dysfunction, a major driver of cardiac disease, is mediated by exosomes (93, 94). During pregnancy, hypoxia triggered exosome signaling increases placental vasculogenesis and augments cytotrophoblastic invasiveness and proliferation as adaptive mechanisms to protect the fetus from oxidative stress (95, 96). In addition, in metabolic disorders such as obesity, exosomes derived from hypoxic adipocytes show an enrichment of lipogenic proteins modulating lipogenic pathways in neighboring adipocytes and pre-adipocytes, thereby transferring characteristics of adipocyte dysfunction (97).

In addition to oxygen tension, the biogenesis and release of exosomes is also affected by glucose concentration. Investigation of the effects of glucose on exosome release showed elevated number of exosomes from trophoblast cells cultured under both high and low glucose concentration (98, 99). Furthermore, the released exosomes induced secretion of pro-inflammatory cytokines from endothelial cells (99). This mechanism potentially mediates the maternal pro-inflammatory profile seen in pregnancies with glucose intolerance. Comparison analysis of plasma exosomal miRNA showed upregulation of miR-326 in diabetic patients compared to controls and this increase negatively correlated with its target, adiponectin (100).

However, the exact mechanism of these alterations in exosome biogenesis and of exosomal miRNA profile under different extracellular glucose concentration is not completely understood. The current body of data suggests that changes in intracellular Ca^2+^ concentration may play a vital role in membrane trafficking, fusion, and retrieval and has intriguing roles in modulating exosome release in response to extracellular glucose (101–103).

## The Human Placenta

A healthy pregnancy outcome is highly reliant on tight physiological regulation that is largely orchestrated by an extremely complex and multifunctional materno-fetal organ, the placenta (104). The human placenta is made up of trophoblast cells specifically the cytotrophoblast, syncytiotrophoblast (ST), and extravillous trophoblast (EVT). The ST cells are in direct contact with the maternal circulation (105). Meanwhile, EVT are a specific type of cells with a high invasive capacity; these cells migrate to the maternal tissue to remodel the uterine spiral arteries (106).

The placenta is a highly multifunctional organ. It regulates the exchange of respiratory gases, provides protection for the fetus against maternal immunity, and removes carbon dioxide and excretions from the fetus through the mother. Furthermore, the human placenta acts as a nutrient sensor, controlling maternal–fetal nutrient transport (107, 108). It detects maternal–fetal nutrient status and alters nutrient transporter capacity to align to fetal growth and nutrient requirements (109, 110). In addition, the placenta is a transient endocrine organ secreting various hormones and cytokines that can directly affect both maternal and fetal metabolism.

### Placenta in Obesity

Cytokines and hormones play major roles in the initiation and preservation of pregnancy. However, the endocrine functions of placenta are greatly affected by maternal obesity. Maternal metainflammation produces signals opposing the normal regulatory functions of the placenta and contributes to the adverse outcomes observed in obese pregnant mothers. The increase in maternal BMI has been positively correlated with an increase in placental weight (111). A population-based study showed that obese pregnant women had higher placental weight with higher plasma glucose and leptin than their non-obese counterparts at term (112).

Obese pregnancies have a dysregulated maternal cytokine profile with a significant rise in pro-inflammatory cytokines (113, 114). In addition to changes in the plasma, changes to the inflammatory profile of the placenta are also observed in obese pregnancies. An increase in TNF-α turnover in obesity is a well-known phenomenon. Similarly, reports of a significant elevation of TNF-α in the circulation and placenta of obese mothers are consistent (115–119). The placental production of leptin leads to maternal hyperleptinemia with downregulation of placental leptin receptors and resultant leptin resistance in obese mothers (120–122). The analysis of placentae from obese mothers also showed increases in other pro-inflammatory cytokines, such as interleukin (IL)-1β and IL-6 (115, 117). A sequencing study of placental RNA highlighted that levels of IL-12Rβ2, IL-21R, and CX3CR1 were increased while IL-R1, IL-1RAP, CXCR1, CXCR2, CCR3, and ADIPOR1 gene were decreased in placentae of obese women (123).

As a whole, obesity in pregnancy has profound effects, causing systemic inflammation. The increase in circulating pro-inflammatory cytokines from adipose tissue may provoke increased inflammatory cytokines secretion by the placenta and alter placental function. The obesity associated with GDM may have similar or enhanced negative consequences for the placenta.

### Placenta in GDM

Pathologically, GDM is characterized by the onset of glucose intolerance of variable severity that is first recognized during pregnancy (124) and a fasting glycemia level ≥92 mg/ml (125). An increase in IR is commonly due to changes in pregnancy-related hormones that occur during early gestation (126). The mother’s inability to secrete sufficient insulin to counteract the IR induced by the gluconeogenic placental hormones may cause the development of GDM (127).

The human placenta is at the materno–fetal interface. Due to its position, the placenta is greatly exposed to various adverse intrauterine conditions and can easily be affected by any changes in its milleu. Glucose is the primary placental energy substrate. Materno–fetal glucose exchange is vital for fetal survival and is observed throughout pregnancy. The gestational changes in maternal glucose metabolism and increased blood glucose level reflect the maternal metabolic adaptations to fulfill the nutrition requirements of the developing fetus. However, this phenomenon is exacerbated in GDM.

The hyperglycemic condition affects trans-placental glucose transport and dysregulation of GLUT activity. In GDM pregnancies, the expression of GLUT1 at the basal membrane was increased twofold with a 40% increase in glucose uptake (128). GLUT1 and mTOR signaling were significantly increased in placentae from GDM pregnancies when compared to normal pregnancies. Interestingly, these changes were associated with a 50% reduction in mitochondrial respiration in trophoblast cells isolated from GDM placentae when compared to the control (i.e., cells from normal placentae) (129). Similarly, utilizing GDM placental explants, a study demonstrated a twofold to threefold increase in glucose uptake (130).

Interestingly, the overexpression of pro-inflammatory cytokines seen in obesity is also observable in GDM placenta. The prominent increase in TNF-α seen in obese pregnancies has also been observed in the maternal circulation and placenta in GDM. The overexpression of TNF-α in GDM placenta is associated with increased fetal adiposity (131, 132). Similarly, Kuzmicki et al. (133) and Lepercq et al. (131) reported an increased IL-8 and leptin expression in GDM placenta, respectively.

The current body of literature suggests that maternal inflammation leads to the over-production of inflammatory cytokines by the placenta that would normally be expressed at significantly lower levels in healthy pregnancies. It is proposed that this enhanced inflammation is associated with the metabolic changes seen in GDM pregnancies. Although these data demonstrate an interaction between maternal obesity and the development of GDM, strikingly, the underlying mechanism that could explain why obesity-associated inflammation is transferred or enhanced in obese-GDM placenta is not understood. Therefore, it can be postulated that other factors mediate the development of GDM by influencing placental function.

### Placental Exosomes in Understanding Pregnancy Pathologies

Besides secreting hormones and cytokines, the placenta extrudes large quantities of EVs (Table 1) constitutively throughout gestation originating mainly from the syncytiotrophoblastic layer (134, 135). EVs, especially exosomes, are packed with a vast repertoire of proteins, miRNAs and phospholipids that play crucial roles in maintaining feto–maternal communication for healthy pregnancy outcomes (136). These exosomes can be identified through their molecular features. In particular, human placental alkaline phosphatase (PLAP) is an allosteric enzyme synthesized in the placenta. Exosomes isolated from the circulation of pregnant women carried PLAP on their membranes; hence, a PLAP^+^ phenotype can be used to identify placental origin (137, 138).

**Table 1:** Summary of studies of EVs derived from placental experimental designs.

EV types	Sample types	Isolation method	Findings	Reference
Exosomes	Plasma	Centrifugation	miRNAs are released *via* exosomes	Luo et al. (79)
STMB	Plasma	Centrifugation	Presence of high level of EVs in late onset preeclampsia	Dragovic et al. (142)
Exosomes	Plasma	Centrifugation + density gradient	Placental exosomes increase from 6 to 12 weeks	Sarker et al. (135)
Exosomes	Plasma	Centrifugation + density gradient	Presence of high levels of placental exosomes in preeclampsia	Pillay et al. (232)
Exosomes	Plasma	Centrifugation + density gradient	Exosome profile changes with gestation change	Salomon et al. (137)
STBM	Plasma	Time-resolved fluoroimmunoassay	STBM increase in preeclampsia	Knight et al. (233)
Exosomes	Plasma	Centrifugation + density gradient	Presence of high levels of placental exosomes in GDM	Salomon et al. (144)
Exosomes	Plasma	Centrifugation + density gradient	Exosome concentration increases with maternal BMI and induce the release of cytokines from the endothelial cells	Elfeky et al. (145)
EVs	Primary trophoblast cells	Centrifugation	Protein and mRNA profile varies between different classes of EVs and possess antiviral activity	Ouyang et al. (136)
Exosomes	Primary trophoblast cells and villous explant	Centrifugation + density gradient	Exosomes modulate maternal immune response	Kshirsagar et al. (140)
Exosomes	Villous explants Primary trophoblast cells and BeWo cells	Centrifugation + density gradient	Exosomes modulate trophoblast syncytium formation	Tolosa et al. (141)
Exosomes	Primary trophoblast cells	Centrifugation + density gradient	Hyperglycemia induces release of exosomes and alters their bioctivity	Rice et al. (99)
Exosomes	Primary trophoblast cells	Centrifugation + density gradient	Under hypoxia exosomes mediate trophoblast migration	Salomon et al. (95)
Exosomes	Primary trophoblast cells JEG-3 BeWo	Centrifugation + density gradient	C19MC is the predominant miRNA species from placenta	Donker et al. (76)
Exosomes	BeWo cells	Centrifugation + density gradient	Differential expression of C19MC in GDM	Almohammadi et al. (78)
STBM	Villous explant	Ultracentrifugation	Protein profile is different in preeclampsia	Baig et al. (234)
STBEV	Dual placental perfusion	Ultracentrifugation	Platelet activating ability of EVs in preeclamsia	Tannetta et al. (235)
STBM	Primary syncytiotrophoblast cells Dual placenta perfusion system	Ultracentrifugation	Pro-inflammatory and anti-angiogenic activity of MVs in preeclampsia	Tannetta et al. (236)
STBM	Dual placenta perfusion system	Ultracentrifugation	Differential expression and pro-inflammatory activity of MV proteins in preeclampsia	Tannetta et al. (237)
STBEV	Dual placenta lobe perfusion model	Ultracentrifugation	Differential enrichment of EVs from placental perfusate	Dragovic et al. (41)
STBM	Dual placenta perfusion system	Ultracentrifugation	Cell-free fetal hemoglobin can change miRNA profile in STBM	Cronqvist et al. (238)

*STMB, syncytiotrophoblast-derived vesicles; EVs, extracellular vesicles; STBEV, syctiotrophoblast-derived extracellular vesicles; MV, microvesicles; GDM, gestational diabetes mellitus*.

Maternal plasma is an excellent source for placenta-derived exosomes with their appearance reported as early as 6 weeks of gestation (138, 139) with concentrations varying in accordance with the stage of gestation (135, 137, 138). The presence of immune molecules such as HLA-G and B7 family in PLAP^+^ exosomes demonstrates their role in maternal immunomodulation. This counteracts allograft rejection of the fetus and sustains cellular adaptation in the face of the physiological changes associated with pregnancy (136, 140). Placenta-derived exosomes carry Synctin-1 that mediates trophoblastic syncytialisation (141) and regulates endothelial cell migration, thereby sculpting the maternal–fetal circulation (137). Thus, the involvement of PLAP^+^ exosomes in various processes, such as immunomodulation and vascular changes, can explain their several fold increase in the early stages of pregnancy (137). In general, the concentration of EVs is higher in pregnancy compared to non-pregnant states (142). Furthermore, the concentration of EVs varies in the presence of pathophysiological conditions such as preeclampsia (143) and GDM (144). Recent evidence suggests that individuals with GDM, in particular, may have a distinct exosomal profile when compared to those from healthy pregnancies. The total number of exosomes in maternal plasma between 11 and 14 weeks of gestation is up to twofold greater in women who later developed GDM (diagnosis at 22–28 weeks) compared to those who had a normoglycaemic pregnancy (144).

In GDM, an environment of hyperglycemic and oxidative stress induces exosome release (99). Interestingly, the elevation in total exosome concentration in maternal plasma significantly correlates with maternal BMI, whereas the ratio of PLAP^+^ to total exosome number decreases with higher maternal BMI across gestation (145). In GDM, the augmentation in exosome numbers is due to an increase in total exosomes other than PLAP^+^ exosomes (144). However, the source of these extra circulating exosomes present in obese and GDM mothers remains unknown.

Hypoxia and elevated glucose concentrations are the hallmarks of GDM, and this alters the exosome profile and bioactivity. Cytotrophoblasts cultured under different oxygen tensions (1, 3, and 8%) showed an increased production of exosomes under low oxygen tension (1%), which promoted increased invasion and proliferation of the cells (95). Co-incubation of exosomes with endothelial cells *in vitro* upregulated the cellular secretion of cytokines. Plasma exosomes isolated from obese and GDM subjects induced the secretion of pro-inflammatory cytokines from endothelial cells from normal and lean pregnancies (144, 145). These findings provide some interesting insights into the role of exosomes in the inflammatory phenomena typically associated with GDM.

Exosomal-mediated miRNA signaling is another fascinating scenario of feto-maternal communication, absolutely essential to maintain the physiological and metabolic harmony between the mother and fetus (79). The dysregulated expression of placental-specific C19MC miRNAs is associated with pathological pregnancies including GDM (146–148). Consistent with this, an increase in the expression of C19MC miRNAs in placental exosomes in the presence of high extracellular glucose was reported (78). Therefore, exosomal miRNA may potentially be involved in placental–maternal signaling.

## Adipose Tissue

Adipose tissue is an inert connective tissue comprised primarily of adipocytes which functions as a fat reservoir. There are two types of adipose tissue, white adipose tissue (WAT) and brown adipose tissue (BAT). Fats are stored as triglycerides and released as free FA whenever the body requires energy. Despite functional differences, the formation of both WAT and BAT is regulated by the process of adipogenesis, which can be divided into two phases. First, this involves the commitment of mesenchymal stem cells (MSC) to becoming preadipocytes followed by the terminal differentiation of preadipocytes into adipocytes (149–152).

Brown adipose tissue is made up of multilocular thermogenic brown adipocytes. The enrichment of iron containing mitochondria and high expression of Uncoupling Protein 1 provides for the thermogenic role of BAT (153). BAT is abundantly present in infants and recent reports demonstrate the presence of functionally relevant BAT in adults (154–156). Interestingly, a high level of BAT activity was associated with low BMI (157, 158). This reflects the probable involvement of BAT in energy metabolism, which is seemingly higher in lean individuals.

On the other hand, WAT is made up of unilocular white adipocytes each containing a single lipid droplet. Besides adipocytes, WAT also comprises stromal cells such as preadipocytes, fibroblasts, macrophages, and endothelial cells (159, 160). Importantly, WAT is involved in energy storage and there are different depots based on its location in the body. Adipose tissue located beneath the skin is known as the subcutaneous adipose tissue, while visceral adipose tissue (VAT) refers to the fat surrounding internal organs. The link between obesity and metabolic disease is most commonly observed with accumulation of VAT.

Besides its function as a thermal regulator and fat-storage site, adipose tissue is the largest endocrine organ and regulates homeostasis by coordinating intercellular communication with other body systems. Adipose tissue readily modulates various biological functions by producing an array of bioactive peptides known as adipocytokines, which are capable of exerting various metabolic effects including those on glucose and lipid metabolism (161–163).

The discovery of leptin gives adipose tissue the status of an endocrine organ. Leptin, the “satiety hormone,” has anorexigenic effects and acts on food intake and fat mass. Leptin, which is involved in energy metabolism, significantly increases in obesity and is present in its free form (164, 165). Adiponectin is an adipose tissue-specific adipokine (166) and is well known for its role in energy homeostasis as well as anti-obesity, anti-inflammatory, and anti-diabetic properties (167–169). Adiponectin promotes glucose utilization and fatty acid oxidation (FAO), which enhances insulin sensitivity (170, 171). Activation of the AMP-activated protein kinase signaling pathway by adiponectin acts as a central regulator of glucose and lipid metabolism (170).

The imbalance between energy intake and expenditure leads to expansion of adipose tissue. The two possible growth mechanisms are hyperplasia and hypertrophy (172). The hyperplastic expansion generates new adipocytes. Meanwhile, hypertrophy beings about an increase in the size of adipocytes (173, 174). The finding that significant weight loss in humans is marked by a reduction in adipocyte volume but not number suggests that adipose tissue hypertrophy is strongly associated with obesity.

## Adipose Tissue in Obesity

Obesity is associated with inflammation, elicited by metabolites which lead to systemic IR. This pro-inflammatory environment in obesity, known as “metainflammation,” (metabolically induced inflammation) is associated with a reduced metabolic rate, maintained by adipose tissue (175). The adipose tissue of obese individuals is known to comprise a greater fraction of fat as the adipose tissue has the ability to adapt to the nutrient environment and store excess energy.

The hypertrophic expansion of adipocytes causes dysregulation of cytokine secretion and is responsible for the low-grade inflammation and several comorbidities seen alongside obesity. In obese individuals, the production of adiponectin decreases with an expansion of the adipose tissues (176). This has been attributed to the failure of transcriptional regulation (177). Hypermethylation of the adiponectin promoter induced by DNA methyltransferase-1 is ascribed to the hypoadiponectinemia seen in obesity (178). The decreased expression of adiponectin is seen in conjunction with effects on glucose metabolism and an increase in IR (176, 179). Besides adiponectin, the expression of adiponectin receptors, ApoR1 and ApoR2, is reduced in obesity, hence enhancing IR (180, 181). Similarly, the abnormal production of leptin in obesity leads to leptin resistance and supresses insulin-stimulated glucose metabolism (182).

In addition, hypertrophic adipocytes secrete elevated amounts of pro-inflammatory cytokines such as TNF-α, IL-6, IL-8, and monocyte chemoattractant protein (MCP) (183–185). The increased secretion of pro-inflammatory cytokines and the relative hypoxia and cell death promoted by hypertrophic adipocytes promotes a high infiltration rate of monocytes into visceral adipose tissue and activation of macrophages (186). Overall, the increase in release of pro-inflammatory cytokines and infiltration of macrophages leads to development of IR (187).

Adipocytokines are known to regulate cellular signaling in various tissues through endocrine mechanisms. However, there is lack of a positive correlation between BMI, adipocytokines, and the development of diabetes in obese pregnancies. In order to further understand these interrelationships, it is necessary to interrogate the potential involvement of adipose tissue-derived exosomes in overall glucose regulation.

### Adipose Tissue in GDM

Maternal body fat mass increases throughout the pregnancy, with accumulation of fat observed on the trunk (188, 189). During pregnancy, appropriate expansion of adipose tissue is vital in order to support nutrient supply to the fetus. However, the hypertrophic growth of adipose tissue is closely associated with metabolic abnormalities and IR (190–192). The ectonucleotide pyrophosphate phosphodiesterase-1 (ENPP-1) is a protein known to induce adipocyte IR. In a recent study, it was demonstrated that adipose tissue from obese patients with GDM expresses high level of ENPP-1 that correlates with the expression of GLUT4 and with insulin receptor substrate-1 serine phosphorylation (193).

Hypertrophy of adipocytes in adipose tissue can impair the functions of adipose tissue, overall. Hypertrophic adipose tissue is associated with excess amount of adiposity and results in a dysregulated secretory profile (194). A higher level of pro-inflammatory cytokines, especially TNF-α and IL-6 has been reported in obese pregnancies (195, 196). The abnormal secretion of adipocytokines is implicated as an essential factor in the development of GDM (197, 198).

Studies to date are suggesting that the relationship between hypertrophic growth of adipose tissue and inflammation is a pivotal factor that causes IR. However, the underlying mechanism by which these adipocytokines affect GDM is not fully understood. While our current understanding of GDM is limited to inflammation induced by adipocytokines, a wide variety of adipose tissue functions may be regulated by adipose tissue-derived exosomes. Therefore, the involvement of adipose tissue-derived exosomes in the development in GDM is possible and understanding of this mechanism is essential.

### Adipose Tissue-Derived Exosomes

In addition to soluble factors, exosomes are also involved in various functions of adipose tissue (Table 2). Adipose tissue-derived exosomes have been isolated from culture medium of adipose tissue, adipocytes, and adipose tissue-derived stem cells (ADSC) (74, 199–202). A recent study demonstrated that both 3T3-L1 adipocytes and primary adipocytes secrete large proportions of exosomes (203). In addition, exosomes secreted by adipocytes were reported to be more abundant compared to exosomes secreted by melanoma cells (204). This suggests the probable participation of adipose tissue/adipocyte-derived exosomes in various biological functions.

**Table 2:** Summary of studies of EVs derived from adipose tissue.

EVs	Source	Isolation method	Findings	Reference
Exosomes	Ad-MSC	Centrifugation	The exosomes showed inhibitory effect in the differentiation and activation of T cells and reduced the proliferation and IFN-γ release	Blazquez et al. (220)
Exosomes	Ad-MSC	Not specified	Graft-versus-host disease patients treated with the exosomes showed reduction in the symptoms	Ludwig et al. (221)
Exosomes	Primary culture of rat adipocytes	Centrifugation + filtration	A total of 509 proteins were identified, some of which are known to express in the adipocytes.Comparison of the exosomes derived from obese diabetic and obese non-diabetic showed differential expression of 200 proteins	Lee et al. (215)
Exosomes and microvesicles	Ad-MSC	Centrifugation	Comparison between MSCs and EVs showed a total of 128 proteins were selectively enriched in the EVs	Eirin et al. (213)
Exosomes and microvesicles	Ad-MSC	Centrifugation	Comparison between MSC and EVs showed enrichment of 4 miRNAs, 255 mRNAs, and 277 proteins enriched in EVs	Eirin et al. (214)
Exosomes	Human adipose tissue	Centrifugation	The exosomes are capable of impairing insulin signaling in the end target organ depending on the contents	Kranendonk et al. (207)
Exosomes and microvesicles	3T3-L1 cells	Centrifugation	The concentration of EVs was higher, pre-adipogenesis and the exosomal proteins content differ between pre- and post-adipogenesis EVs	Connolly et al. (13)
Exosomes	Mice visceral adipose tissue	Centrifugation + density gradient	The exosomes released from obese adipose tissue induced the differentiation of monocytes to macrophages and development of insulin resistance in lean mice	Deng et al. (74)
Exosomes	Ad-MSC	Centrifugation	The exosomes promoted migration and upregulation of cancer-related signaling pathways in MCF7	Lin et al. (201)
Exosomes	3T3-L1	Commercial kit	The exosomes reduced the accumulation of mHtt aggregates, improved mitochondrial dysfunction, and increased the survival of the cells	Lee et al. (217)
Exosomes and microvesicles	3T3-L1 Primary culture of rat adipocytes Plasma	Centrifugation	Perilipin A is enriched in adipocyte-derived EVs, especially from obese adipocytes. The expression decreased with reduced calorie diet intervention	Eguchi et al. (212)
Exosomes	3T3-F442A Mice adipose tissue	Centrifugation + density gradient	The exosomes promoted the migration of the tumor cells through fatty acid oxidation	Lazar et al. (204)
Exosomes and microvesicles	Ad-MSC	Filtration + centrifugation	The EVs decreased the apoptosis of the neuronal cells and increased remyelination and activation of neuroglial precursors	Farinazzo et al. (218)
Exosomes	Ad-stromal cells	Commercial kit	The exosomes protected the NSC-34 cells from oxidative damage and increased their survival	Bonafede et al. (219)
Exosomes	Ad-MSC	Commercial kit	The miR-122 in the exosomes increased the sensitivity of the hepatocellular carcinoma cells to chemotherapeutic agents	Lou et al. (18)
Exosomes	Human adipose tissue	Commercial kit	The miRNAs were differentially expressed between lean and obese exosomes and the obese exosomes induced TGF-β pathway dysregulation in HepG2 cells	Koeck et al. (200)
Exosomes	SGBS Human adipose tissue	Centrifugation + density gradient	The exosomes differentiated the monocytes into macrophages. The macrophages pre-treated with exosomes from adipose tissue inhibited Akt-phosphorylation and insulin resistance in adipocytes	Kranendonk et al. (222)
Exosomes	Human adipose tissue	Commercial kit	The exosomes from obese adipose tissue suppressed the phosphorylation of Akt in both lean and obese skeletal muscle	Park et al. (14)
Exosomes	Human adipose tissue	Commercial kit	Differentially expressed miRNAs between lean and obese adipose-derived exosomes targets the TGF-β signaling and Wnt/β-catenin signaling pathways	Ferrante et al. (205)
Exosomes	Urine Serum	Not specified	The exosomes contained mRNAs targeting the TGF-β signaling which is associated with airway remodeling	Epstein et al. (228)
Exosomes	Plasma Serum	Commercial kit	The miRNA profile of exosomes changed subsequent to gastric bypass and improved the insulin resistance	Hubal et al. (229)
Exosomes	3T3-L1	Centrifugation + filtration	The hypoxic adipocyte-derived exosomes showed altered expression and increased secretion of proteins compared to normal adipocyte-derived exosomes	Sano et al. (97)

*Ad-MSC, adipose tissue-derived mesenchymal stem cell; EVs, extracellular vesicles; MSC, mesenchymal stem cells; 3T3-L1, preadipocyte cell line; MCF-7, human breast adenocarcinoma cell line; mHtt, mutant Huntington protein; 3T3-F442A, preadipocyte cell line; Ad-stromal cells, adipose tissue-derived stromal cells; NSC-34, motor neuron-like cells; HepG2, human liver cancer cell line; SGBS, Simpson–Golabi–Behmel syndrome preadipocyte cell strain*.

Although most studies report adipose tissue-derived exosomes within the proposed size range of exosomes (203, 205), Katsuda et al. (206) reported ADSC-derived exosomes that were larger. This indicates that the size range of the exosomes may differ based on the cellular source of isolation. In addition to the identification of exosomal markers, adipose tissue-derived exosomes can be characterized based on the presence of adipose tissue-specific markers, such as fatty acid binding protein 4 (FABP4; adipocyte differentiation marker) and adiponectin (205, 207, 208).

Interestingly, the characterization of exosomes released pre- and post-adipogenesis showed differences in the protein content. Pref-1 and FABP4 were decreased while adiponectin was increased in the post-adipogenesis exosomes. However, there were no changes in the exosomal markers, such as CD9, CD63, TSG101, and Alix (13). This shows that proteins, which are commonly used for bio-marking exosomes, can be used to identify the adipose tissue-derived exosomes.

The release of exosomes has been reported to vary depending on body weight. The concentration of exosomes differs between adipose tissue from lean and obese individuals. The quantification of exosomes isolated from subcutaneous and omental ADSC of lean and obese donors showed that ADSC from obese individuals secretes higher concentrations of exosomes (202). Similarly, the primary adipocytes of obese animals secreted more exosomes when compared to lean animals (204). Surprisingly, exosomes isolated from adipose tissue explants derived from lean and obese individuals showed a higher number of exosomes released by lean adipose tissue (205). The secretion of a higher amount of exosomes by lean adipose tissue may be attributed to the fact that there is a higher number of adipocytes in lean adipose tissue. However, a larger proportion of adipose tissue in obese individuals may explain the isolation of higher number of exosomes from obese individuals (205). In addition, the release of adipose tissue-derived exosomes is influenced by the extracellular milieu. Adipose tissue hypoxia is one of the dysfunctional processes seen in adipose hypertrophy which can cause dysregulated secretion of adipocytokines (209). 3T3-L1 adipocytes cultured under hypoxic conditions released a higher amount of exosomes (97). Thus, the nature or condition of their parent cell determines exosome secretion.

#### Composition of Adipose Tissue-Derived Exosomes

The contents of adipose tissue-derived exosomes are similar to their parent cell. The characterization of adipose tissue-derived exosomes demonstrated the presence of various adipocytokines, such as adiponectin, leptin, resistin, TNF-α, and various ILs that can be found in the adipose tissue (210). Adipose tissue-derived exosomes are also enriched with enzymes, such as acetyl-CoA carboxylase, glucose-6-phosphate dehydrogenase, FA synthase, and lipids (13, 97, 211). Interestingly, the levels of these enzymes were found to be upregulated in obesity (97). Comparison of the circulating vesicles from adipose tissue before and after a reduced calorie diet intervention showed a decreased enrichment of perilipin-A in the vesicles (212). Thus, analysis of the composition of adipose tissue-derived exosomes will be an effective reflection of the metabolic state of the adipose tissue.

Besides reflecting their parent cell, the contents of exosomes act as an important component in coordinating functions and influencing the behavior of the end target cells. In addition, their contents can reflect the microenvironment of the exosomes. In relation to this, the analysis of the composition of overall adipose tissue MSC-derived EVs showed selective enrichment of 128 proteins compared to the adipose tissue MSC (213). Another study demonstrated selective enrichment of 4 miRNAs, 255 mRNAs, and 277 proteins enriched in these EVs (214). Exosomes isolated from hypoxic conditions showed upregulated expression of lipogenic enzymes (97). The proteomic analysis of adipose tissue-derived exosomes isolated from obese-diabetic and obese-non-diabetic rats showed the presence of 509 proteins. Among these proteins, 200 proteins were dysregulated in exosomes isolated from adipose tissue of obese-diabetic rats (215). The dysregulated proteins have been shown to be similarly dysregulated in T2D (215, 216). The changes in proteomic content of adipose tissue-derived exosomes reflect the condition of obesity and its related comorbidities. Therefore, characterization and quantification of the contents of the exosomes will provide insight into the health status of the adipose tissue and reflect their involvement in various biological functions.

#### Biological Properties of Adipose Tissue-Derived Exosomes

Adipose tissue-derived exosomes are heterogeneous in function and act in both an autocrine and a paracrine manner. Based on these roles, recent findings demonstrate that adipose tissue-derived exosomes may be an underlying mechanism for the regulation of various biological functions and progression of various diseases.

The treatment of the Huntington’s disease cell line with ADSC-derived exosomes reduced the mHtt aggregates and saved the cells from apoptosis (217). The exosomes were also shown to be involved in nerve regeneration. The exosomes inhibited neuronal cell death and promoted re-myelination and re-genesis of neurons (218). In addition, the exosomes increased the viability of the neuron-like cells expressing amyotrophic lateral sclerosis mutation (219). Hence, adipose tissue-derived exosomes have complex functions in the regulation of nerves and neurons, and more broadly, are implicated in progression disease states. This is also supported by the role of adipose tissue-derived exosomes in immune regulation. Exosomes from ADSC impaired the proliferation rate of T cells and inhibited the activation by reducing the secretion of IFN-γ (220). Meanwhile, the exosomes from MSC temporarily improved the symptoms in graft-versus-host disease patients (221). Overall, the current body of literature highlights multifaceted roles for adipose tissue-derived exosomes and multiple key areas in which these exosomes regulate biological function.

Adipose tissue-derived exosomes have been reported to promote tumor growth. The treatment of the MCF7 breast cancer cell line with exosomes derived from ADSC showed greater migration *via* activation of the Wnt signaling pathway (201). The melanoma cells incubated with exosomes secreted by the 3T3-F442A cells exhibited enhanced migration capacity. The exosomes, which were enriched with trifunctional enzyme subunit α, mitochondrial and hydroxyacyl-coenzyme-A-dehydrogenase, escalated FAO regulating tumor progression (204). Given the involvement of adipose tissue-derived exosomes in the progression and development of tumors, targeting this will be an essential aspect in cancer treatment. Cancer therapy is a key area of interest, as adipose tissue-derived exosome have also been used as carriers of specific cargo. Exosomes from miR-122 transfected adipose tissue MSC showed expression of the miRNA. The exosomes then delivered the miRNA to carcinoma cells, increasing their sensitivity to chemotherapeutic agents (18).

Similar to adipose tissue, adipose tissue-derived exosomes are involved in metabolic regulation. Incubation of monocytes with adipocyte-derived exosomes resulted in differentiation of the monocytes into macrophages with upregulation of pro-inflammatory genes. The macrophages also inhibited phosphorylation of Akt in the adipose tissue (222). The inflammatory role of adipose tissue-derived exosomes is exaggerated in obesity and plays a major role in development of obesity-related diseases, mainly in systemic IR. The injection of adipose tissue-derived exosomes into mice showed uptake by monocytes, differentiation into activated macrophages, and secretion of higher amounts of pro-inflammatory cytokines. In the same study, the C2C12 culture treated with adipose tissue-derived exosomes-conditioned media showed impaired activation of the insulin response (74). The exosomes derived from obese adipose tissue suppressed Akt-phosphorylation in both lean and obese skeletal muscle cells.

Intriguingly, the effects of adipose tissue-derived exosomes can be cell specific. Stimulation of HepG2 and C2C12 cells with adipose tissue-derived exosomes caused IR by inhibiting Akt-phosphorylation. However, this effect was more prominent in the liver cells. Furthermore, the adipocytokine content of the adipose tissue-derived exosomes determined the degree of Akt inhibition (207). The activation of transforming growth factor (TGF)-β is related to the development of NAFLD (223). Co-incubation of exosomes from obese adipose tissue-derived exosomes with HepG2 cells caused hyperstimulation of TGF-β (200).

Overall, these data support a role of adipose tissue-derived exosomes in mediating signaling in the end organ. Since the regulation of the signaling pathways are mediated by miRNAs, profiling of adipose tissue-derived exosome miRNA contents is essential in further understanding in this unique mode of cell–cell communication.

#### Adipose Tissue-Derived Exosomal miRNA

Exosomal miRNAs have been identified as novel and promising biomarkers for the diagnosis and prognosis of various diseases. miRNA in adipose tissue-derived exosomes plays a major role in regulating gene expression in adipose tissue as well as in distant cells. A previous study showed the presence of 143 adipocyte-specific miRNAs in adipose tissue-derived exosomes isolated from mice adipose tissue (199). In a recent study, the profiling of adipose tissue-derived exosomes isolated from mice serum detected 653 miRNAs. In addition, fat transplantation from wild-type mice to ADicer knock out mice showed restoration of approximately 50% of circulating miRNAs (224). This shows that miRNAs in adipose tissue-derived exosomes contribute to a large amount of circulating exosomal miRNAs. This also points to involvement in regulating various biological functions in adipose tissue and distant cells.

The miRNAs involved in adipogenesis are among the abundant miRNAs in adipose tissue-derived exosomes. Among the adipogenic miRNA found in adipose tissue-derived exosomes are miR-103, miR-146-b, and miR-148-a (225–227). However, obesity and its related diseases influence the expression of exosomal miRNAs. The profiling of the adipose tissue-derived exosomes isolated from lean and obese individuals showed the differential expression of 88 miRNAs with significant upregulation of miR-23-b and miR-4429. These miRNAs were shown to activate the TGF-β and Wnt/β-catenin signaling pathways in the end target organs, causing obesity-related conditions (205). Adipose-derived exosomes isolated from serum and urine of obese youths with physician-diagnosed asthma showed differential expression of miRNAs (miR-15a-5p, miR-153-3p, and miR-138-5p) which target TGF-β signaling and is associated with poor asthma outcome (228). Adipose tissue exosomal miRNAs are also associated with development of IR. The analysis of adipose tissue-derived exosomal miRNA content pre- and post-gastric bypass showed upregulation of miR-103-3p which is known to target the insulin receptor signaling pathway and was previously found to be downregulated in diabetes (229–231).

These studies demonstrate that adipose tissue-derived exosomes and their content can mediate gene regulation and functioning in distant cells. Therefore, in obese pregnancies, adipose tissue-derived exosomes may communicate with the placenta and induce changes in its function which may contribute to the development of GDM. Thus, it is possible that adipose tissue-derived exosomes are the primary factor in the pathogenesis of GDM.

## Conclusion

**Figure 1:**
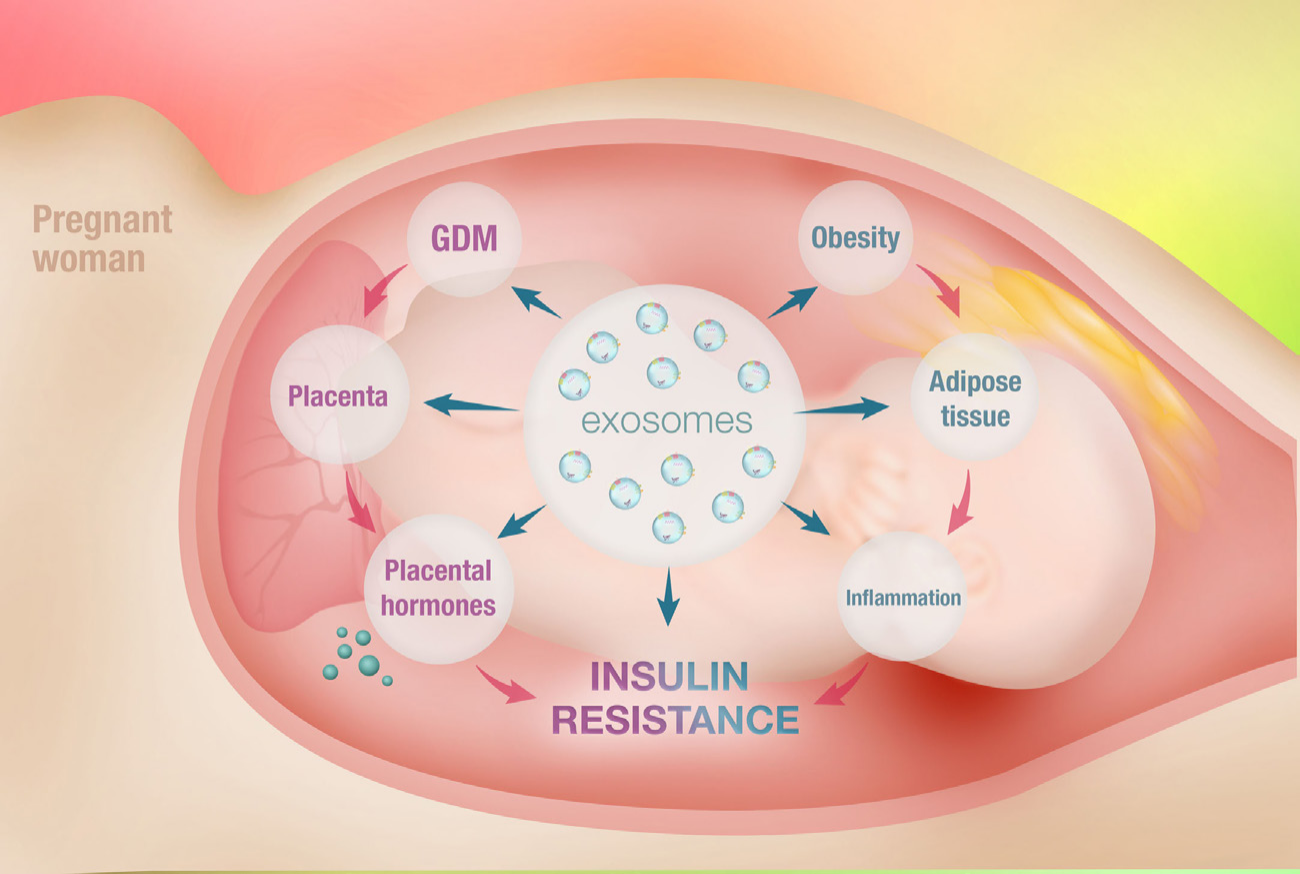
Schematic diagram of intercellular communication between adipose tissue and placenta mediated by adipose tissue-derived exosomes. Obesity refers to an accumulation of excessive fat in adipose tissue due to an imbalance between energy intake and expenditure. This causes hypertrophic expansion of adipocytes and abnormalities in physiological regulation. This is associated with increased free fatty acid release, activation of macrophages, and secretion of elevated amount of pro-inflammatory cytokines, causing systemic inflammation. This is known as metabolically induced inflammation. The marked increase in systemic inflammation is associated with the development of obesity-induced insulin resistance. Gestational diabetes mellitus (GDM) is glucose intolerance diagnosed for the first time during pregnancy. Placental morphological changes as well as altered placental metabolic status are observed in GDM. The placental dysfunction seen in GDM represents an adaptation of the placenta to increased maternal inflammation and results in increased secretion of inflammatory cytokines, further exacerbating inflammation. This potentially causes impairment in insulin sensitivity and development of GDM. However, the evolving concept of maternal obesity and inflammation may not be the full story in the development of GDM. This is due to insufficient data supporting a role for inflammatory cytokines as an initiator of insulin resistance in pregnancy. Interestingly, the various functions of adipose tissue are also orchestrated by the exosomes. Exosomes are mediators of intercellular communication and are capable of regulating cellular mechanisms. Exosomes from adipose tissue are known to regulate the metabolic activity of various cells *via* paracrine mechanisms. In obesity, adipose tissue-derived exosomes cargo profiles are dysregulated and mediate obesity-associated diseases, including insulin resistance. Thus, it is fair to speculate that the adipose tissue-derived exosomes potentially mediate the communication between adipose tissue and placenta, playing an important role in the development of GDM.

Exosomes are currently a prominent research interest owing to their unique role in intracellular communication and signaling. In addition, exosomes transport bioactive molecules, such as proteins, lipids, mRNAs, and miRNAs. Exosomal miRNA is a notable feature of exosomes that results in the transfer of the genetic material from one cell to another. This functional mechanism has important relevance in the pathogenesis of various diseases, particularly obesity and GDM (Figure 1).

The IR seen in obesity is maintained by adipose tissue. The dysregulated secretion of bioactive molecules by hypertrophic adipose tissue contributes to the development of IR in obese patients. Besides adipocytokines, the adipose tissue also releases exosomes, which are known to mediate IR and various metabolic disorders associated with obesity. Obesity is an underlying mechanism for the development of GDM. In addition, adipose tissue-derived exosomes are altered in metabolic disorders. Hence, we can postulate that the dysregulated secretion of adipose tissue-derived exosomes plays a pivotal role in the development of GDM in obese mothers.

Hypertrophic adipose tissue may cause differential expression of exosomal miRNA. This may further contribute to the systemic inflammation and IR seen in obese GDM pregnancies. This may also alter placental metabolism and nutrient uptake status by deregulating the placental nutrient signaling pathways. Overall, investigating the adipose tissue-derived exosomes present in maternal circulation of obese GDM pregnancies will provide a novel approach to further elucidate the pathophysiology of GDM.

## Author Contributions

NJ, SN, ZN, and CS conducted a review of the literature. GER, FZ, LS AL, JG, CSAN, ML and DF critically reviewed the manuscript.

## Conflict of Interest Statement

The author declares that the literature review was conducted in the absence of any commercial or financial relationships that could be construed as a potential conflict of interest.

## Abbreviations

ADSC, adipose tissue-derived stem cells; BAT, brown adipose tissue; BMI, body mass index; C19MC, chromosome 19 miRNA cluster; ENPP-1, ectonucleotide pyrophosphatase phosphodiesterase-1; ESCRT, endosomal sorting complex required for transport; EVs, extracellular vesicles; EVT, extravillous trophoblasts; FA, fatty acid; FABP4, fatty acid binding protein 4; GDM, gestational diabetes mellitus; GLUT, glucose transporter; HIF, hypoxia-inducible factor; HSP, heat shock protein; IFN-γ, interferon-γ; IL, interleukin; IR, insulin resistance; miRNA, microRNA; MSC, mesenchymal stem cells; MVB, multivesicular bodies; MVs, microvesicles; NAFLD, non-alcoholic fatty liver disease; PI3K, phosphatidylinositol 3-kinase; PLAP, placental alkaline phosphatase; ST, syncytiotrophoblast; TGF-β, transforming growth factor-β; TNF-α, tumor necrosis factor-α; VAT, visceral adipose tissue; WAT, white adipose tissue.
